# Physical Functional Characteristics of Elite Adolescent and Collegiate Male Soccer Athletes: A Comparative Study Using Medical Check-Ups

**DOI:** 10.3390/jfmk11010107

**Published:** 2026-03-05

**Authors:** Tingxu Zhang, Hanyan Yan, Ziwen Mu, Ang Ni, Haoxiang Wang, Zhiqiang Han, Kazuhiro Imai, Xiao Zhou

**Affiliations:** 1 School of Physical Education, Huazhong University of Science and Technology, Wuhan 430074, China; m202375457@hust.edu.cn (T.Z.); yan000719@outlook.com (H.Y.); mzw0528@outlook.com (Z.M.); m202475615@hust.edu.cn (A.N.); w1377997634@163.com (H.W.); h2221578393@163.com (Z.H.); 2 Department of Life Sciences, Graduate School of Arts and Sciences, The University of Tokyo, Tokyo 1538902, Japan; imai@idaten.c.u-tokyo.ac.jp

**Keywords:** soccer players, physical function, adolescent, adult

## Abstract

**Background:** Physical functional capacity plays a critical role in sports performance and changes markedly from adolescence to adulthood. This study aimed to compare the physical functional characteristics between adolescent and collegiate soccer athletes. **Methods:** Fifty elite male soccer athletes (30 adolescents, 20 college students) were assessed for joint range of motion, muscle flexibility, dynamic balance, and trunk functional capacity. **Results:** Adolescent athletes achieved significantly greater general joint laxity score than collegiate athletes (*p* = 0.01), with significantly greater hip range of motion across all planes (abduction, internal rotation, and external rotation; all *p* < 0.01). College athletes had significantly lower SLR degree (left: *p* < 0.01, right: *p* < 0.05) but significantly greater degrees on passive Ely’s test (*p* < 0.01) than adolescent athletes. Collegiate athletes delivered significantly superior dynamic balance performance in the Y-balance test, particularly in the posterolateral and posteromedial directions (all *p* < 0.05). Unexpectedly, trunk functional capacity was significantly lower in collegiate athletes compared with adolescents (*p* < 0.01). Limb asymmetry was observed in both groups: collegiate athletes showed asymmetry only in the anterior reach direction of the Y-balance test (*p* = 0.018), whereas adolescents exhibited asymmetry across multiple joints (ankle, hip, hamstrings, and quadriceps; all *p* < 0.05) and in the posterolateral direction of the Y-balance test (*p* < 0.01). **Conclusions:** Adolescent athletes demonstrated significantly superior joint range of motion and lower limb flexibility, whereas collegiate players exhibited better balance performance, indicating distinct functional profiles between the two cohorts, which may be associated with differences in training experience and developmental stages.

## 1. Introduction

Football, as the world’s most widely participated sport, requires athletes to repeatedly execute high-intensity intermittent movements during matches, including sudden acceleration/deceleration, multi-directional cutting maneuvers, jump-landing actions, and confrontational technical activities, forming complex motor patterns [1]. Its specialized characteristics and the physical function of athletes remain critical research topics in sports science. Consequently, to meet competitive demands, athletes need to develop multidimensional physical functional capacities [2]. Traditional theory emphasizes the deterministic roles of three fundamental physical attributes on sports performance, that is, strength, speed, and endurance. However, recent studies confirmed that composite qualities such as flexibility, coordination, and balance were key elements supporting sport-specific technical actions [3,4,5].

Deficiencies in physical functional elements lead to diminished soccer performance: (1) insufficient joint range of motion restricts maximal movement amplitude in actions such as kicking and cutting, resulting in joint stiffness that reduces movement efficiency and increases injury risk [6]; (2) poor muscular flexibility limits motion range in techniques such as kicking leg swing while exacerbating injury risks due to tension imbalances between quadriceps and hamstring muscles [7]; (3) inadequate balance capacity impairs athletes’ ability to maintain stability during single-leg support and rapid directional changes, compromising technical precision [8]; and (4) deficient trunk strength, the foundation of core stability, restricts force transfer between upper and lower limbs, causing movement breakdown during physical confrontations and reducing core power generation efficiency [9,10].

Research on physical function has focused on epidemiology and injury rehabilitation, though it is increasingly being applied to athletic training. Previous studies demonstrated that medical screening-based assessments of athletes’ functional characteristics could effectively identify deficits and provide actionable insights for coaching strategies [11]. Furthermore, researchers noted significant age-related differences in physical functional attributes among athletes [12]. Such age-specific characteristics have been observed across multiple sports: straight leg raise (SLR) testing in basketball players of different age groups has revealed marked differences in hamstring strength [13]; tennis players across age categories have demonstrated significant differences in performance on the Y-balance test [14]; and the likelihood of restricted lower-extremity range of motion among adolescent baseball players varied according to age group [15]. The differences in age lead athletes to exhibit distinct physical functional characteristics during sport participation, and the application of uniform assessment criteria across different age groups may introduce validity bias. Therefore, age-specific investigations of physical function warrant greater attention in soccer. At present, research examining the physical functional characteristics of soccer players across different age groups in China remains limited. Accordingly, this study aimed to compare the physical functional characteristics of two distinct cohorts of elite soccer athletes by age and hypothesized that joint range of motion, muscle flexibility, balance ability, and trunk function would differ between age groups.

## 2. Materials and Methods

### 2.1. Participants

This study recruited 51 male soccer players, comprising 31 adolescent players from Henan Provincial Experimental High School and 20 collegiate players from Huazhong University of Science and Technology (all having competed in provincial-level or higher competitions with top-eight finishes). Ultimately, one participant was excluded due to failure to complete the physical function test resulting from physical discomfort (Table 1). Consequently, collected data from 50 athletes were used for final analysis. This study was reviewed and approved by the Institutional Ethics Board of Tongji Medical College, Huazhong University of Science and Technology, China (Notification Number [2023] IEC (S172)).

### 2.2. Questionnaire

Via questionnaire-based collection, this study documented the following basic characteristics: (1) age, (2) height, (3) weight, (4) preferred foot (footedness), (5) competitive level, (6) training duration, (7) athletic experience (including training frequency and years), and (8) injury history.

### 2.3. Procedure

#### 2.3.1. Instruments

**Figure 1:**
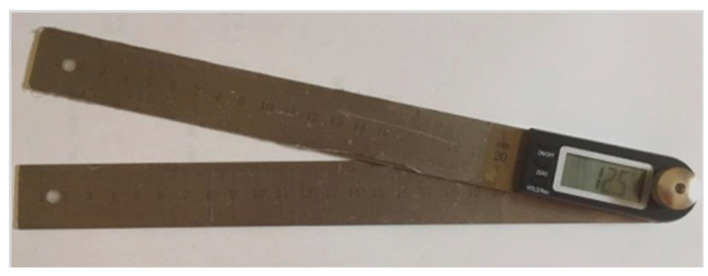
Goniometer.

A goniometer consists of a stationary arm (a straight ruler), a rotating movable arm (another straight ruler), and a central axis (digital goniometer SA-5468, measuring range is 0–360.0 degree with a sensitivity of 0.1 degree with resolution of 0.05 degree, Suncosmo, Tokyo, Japan) (Figure 1).

**Figure 2:**
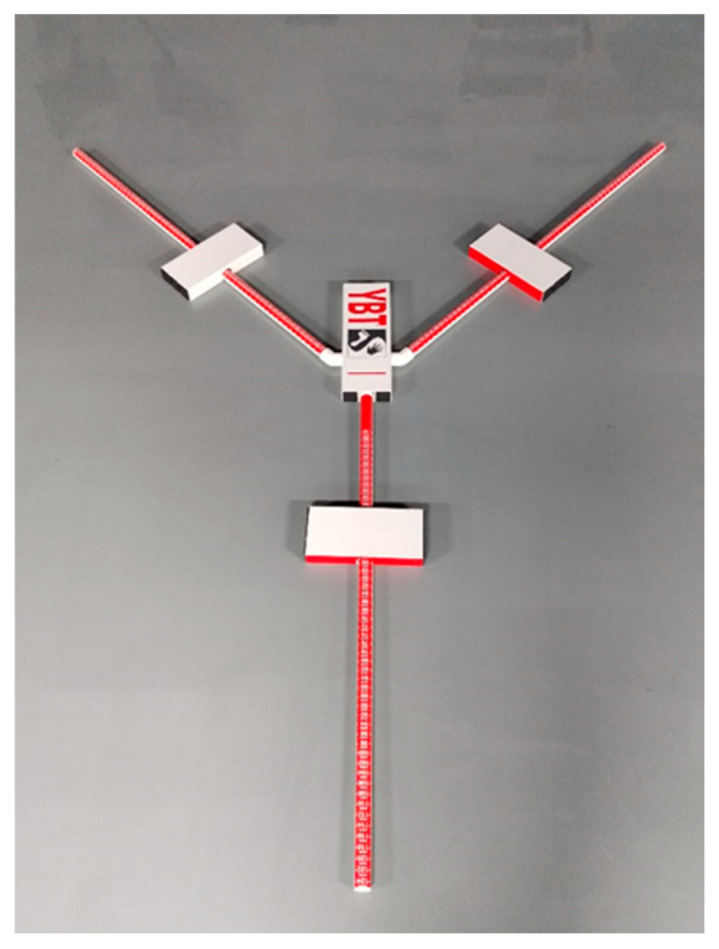
Y-balance test kit.

The Y-balance test kit (WanNiu Fitness Equipment Co., Ltd., Jinan, China) is a dynamic balance assessment tool used to evaluate athletic or clinical populations regarding motor function, injury risk, and rehabilitation progress (Figure 2) by measuring the maximum reach distance in three directions (anterior, posteromedial, and posterolateral) while standing on one leg.

A handheld timer was used to measure time during the single-leg stance with eyes closed and trunk extension tests, with a minimum recorded time unit being milliseconds (YS-802, YingSheng Technology Co., Ltd. Shenzhen, China).

#### 2.3.2. Preparation

Physical function tests were administered to 31 athletes at the Henan Provincial Experimental High School football training facility on 12–13 April 2024 and to 20 athletes at the Sports Training Laboratory of Huazhong University of Science and Technology between October and November 2024. Before testing, participants performed a 10 min standardized warm-up protocol to meet readiness requirements. Examiners provided brief task familiarization to participants and ensured device standardization through rigorous inspection and calibration to guarantee procedural integrity. All participants were instructed to maintain their normal diet during the testing week, fast for at least 2 h before testing, and abstain from depressants (e.g., alcohol) or any known stimulants (e.g., caffeine) within 24 h preceding the assessment. The participants refrained from any training sessions immediately prior to measurement. To minimize measurement variability attributable to factors such as ambient temperature and dietary timing, all testing sessions were conducted indoors within a controlled temperature environment between 14:00 and 17:00. All assessments across both institutions were conducted by the same team of researchers.

#### 2.3.3. Test Process

**Figure 3:**
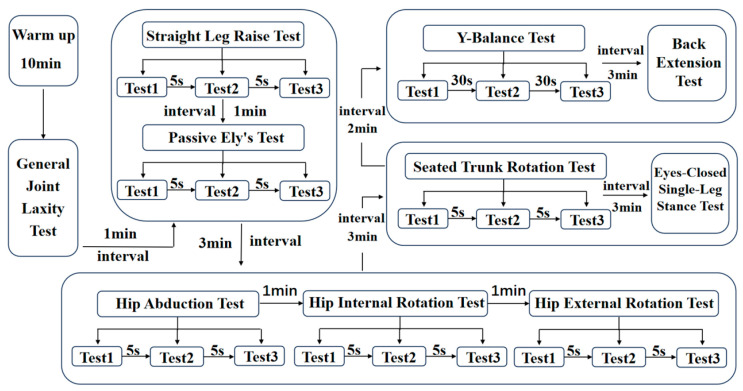
Test flowchart.

This flowchart illustrates a warm-up and physical function test protocol with a total duration of approximately 30 min (Figure 3). The procedural steps were as follows:

1. Warm-Up Activities: The following lower-body foundational warm-up is designed according to the National Strength and Conditioning Association (NSCA) principles:
  ➀ Hip Activation and Dynamic Stretching:
    (a) Walking Forward Lunge: 8–10 steps per side, emphasizing hip flexion/extension range.
    (b) Lateral Lunge: 6–8 repetitions per side, activating adductors and hip abductors.
    (c) Spider-Man Crawl: 4–6 repetitions per side, simultaneously stretching hip flexors and hamstrings.
  ➁ Knee Stability and Ankle Mobility:
    Knee-to-Chest Hold + Ankle Circles: Single-leg stance while pulling the knee to the chest and holding with both hands. Maintain balance while performing ankle circles (5 s clockwise + 5 s counterclockwise per direction). Repeat 2–3 times per side.
  ➂ Dynamic Hamstring and Calf Stretch:
    Walking Straight-Leg Kicks: Alternately kick legs forward while walking, maintaining knee extension. Point toes towards the ceiling. Perform 8–10 kicks per side.
  ➃ Agility and Coordination Drill:
    Rapid Alternating High Knees (Stationary): Quick, alternating knee lifts emphasizing elastic foot contact and ground contact frequency. Duration: 20–30 s. Repeat for 2 sets.
2. General Joint Laxity Test
3. Straight Leg Raise (SLR) Test
4. Passive Ely’s Test
5. Hip Abduction Test
6. Hip Internal/External Rotation Test
7. Seated Trunk Rotation Test
8. Eyes-Closed Single-Leg Stance Test
9. Y-Balance Test (YBT)
10. Back Extension Test

#### 2.3.4. Items

1. General Joint Laxity Test

**Figure 4:**
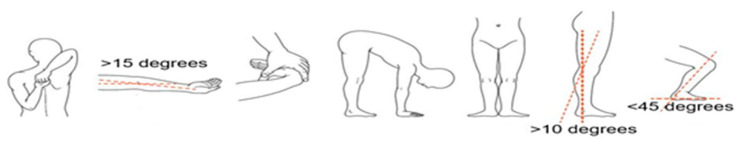
General joint laxity test.

General joint laxity test evaluated seven criteria (Figure 4): (1) excessive shoulder external rotation: defined as the participant’s ability to clasp hands behind the back (hands-behind-back clasp test); (2) elbow hyperextension: ≥15° extension beyond neutral; (3) passive thumb opposition to forearm: the ability to passively abduct the thumb to touch the volar aspect of the ipsilateral forearm; (4) excessive trunk forward flexion: defined as the ability to place both palms flat on the floor with knees fully extended (standing forward bend test); (5) excessive hip external rotation: defined as ≥180° combined external rotation angle of the hips while standing with feet together and toes pointed outward; (6) knee hyperextension: ≥10° extension beyond neutral; and (7) ankle dorsiflexion: ≤45° dorsiflexion measured during passive knee flexion. Scoring: one point was awarded for each criterion where the range of motion met or exceeded the defined threshold. The wrist, elbow, shoulder, knee, and ankle tests were scored per side (0.5 points per side). The maximum total possible score was 7 points [16].

2. Straight Leg Raise (SLR) Test

**Figure 5:**
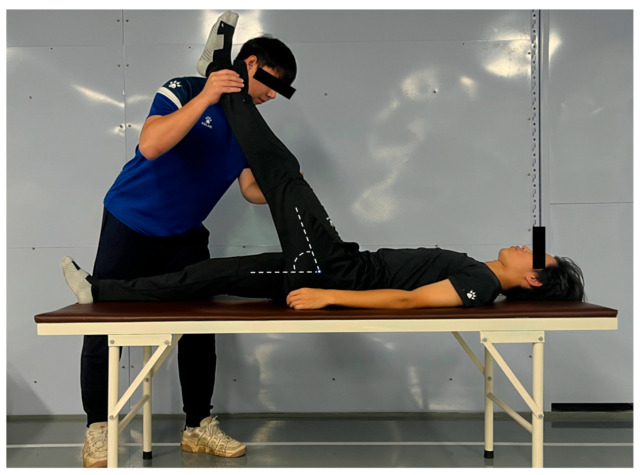
Straight leg raise (SLR) test.

The participants assumed a supine position on a plinth with both legs in a neutral resting position. The examiner passively elevated one test leg while ensuring: (1) knee extension in both legs and (2) flexion of the contralateral hip to prevent movement. The test leg was raised maximally until one of the following endpoints was reached: (1) passive resistance threshold: the examiner applied maximal effort without inducing further leg displacement or (2) participant-reported endpoint: active cessation due to significant pain or discomfort signaled by the participant. At the terminal position, the angle of hip flexion (relative to the horizontal plane) was measured and recorded using a goniometer (Figure 5). The procedure was repeated bilaterally, with three trials per limb separated by 5 s rest intervals. This standardized measurement protocol assessed hamstring flexibility. intraclass correlation coefficient (ICC) for inter-trial reliability = 0.88 [17].

3. Passive Ely’s Test

**Figure 6:**
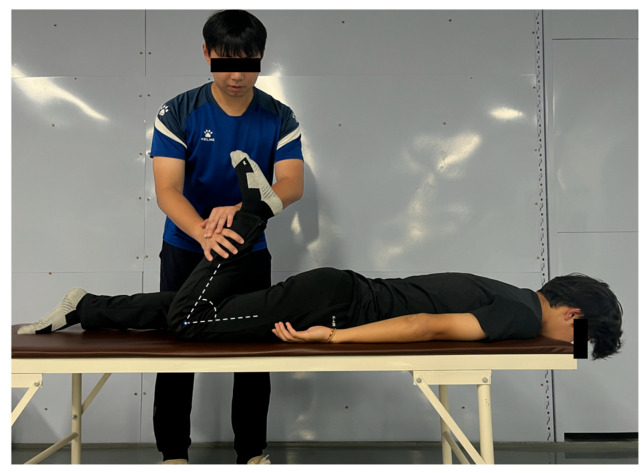
Passive Ely’s test.

The participants assumed a prone position on a plinth with one knee flexed and the contralateral limb in a neutral resting position. The examiner applied downward pressure to the posterior aspect of the ankle of the test limb to achieve maximal passive knee flexion (minimizing the thigh–calf angle) (Figure 6). At the terminal position, the knee flexion angle was measured and recorded using a goniometer. The testing was performed bilaterally with three trials per limb separated by 5 s rest intervals. The procedure assessed quadriceps flexibility, terminating at one of the following endpoints: (1) passive resistance threshold: examiner-applied maximal force without further limb displacement or (2) participant-reported endpoint: voluntary cessation due to significant pain or discomfort (intraclass correlation coefficient, ICC) = 0.914 [18].

4. Hip Abduction Test

**Figure 7:**
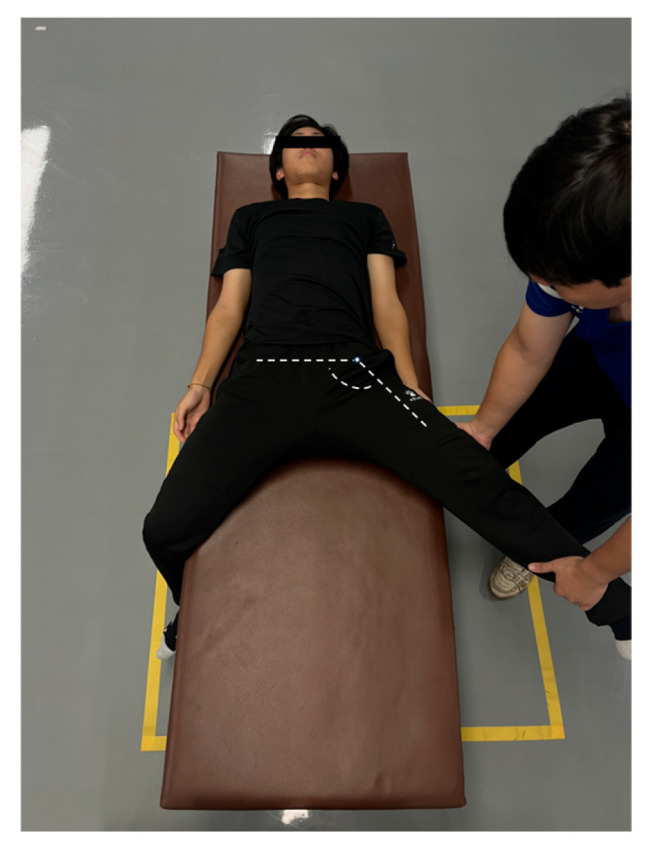
Hip abduction test.

The participants assumed a supine position on a plinth with the non-test leg hanging freely over the edge and the test leg in an anatomically neutral position. The examiner passively abducted the test leg while maintaining: (1) trunk stabilization and (2) knee extension (Figure 7). The terminal hip abduction angle was recorded using a goniometer. The testing was performed bilaterally with three trials per limb (30 s inter-set rest periods) to assess the hip abductor function. The procedure terminated at either endpoint: (1) passive resistance threshold: examiner-applied maximal force without further limb displacement or (2) participant-reported endpoint: voluntary cessation due to pain/discomfort. Excellent inter-trial reliability was demonstrated (ICC = 0.87–0.92) [19].

5. Hip Internal/External Rotation Test

**Figure 8:**
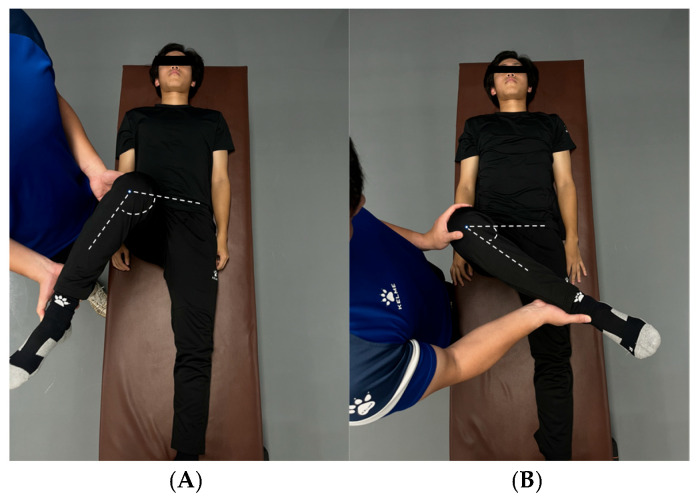
Hip rotation test. (**A**) Hip internal rotation test; (**B**) hip external rotation test.

The participants assumed a supine position on a plinth with the test hip and knee flexed to 90°. The contralateral limb remained in an anatomically neutral position. The examiner passively externally (Figure 8A)/internally (Figure 8B) rotated the test leg’s lower leg while maintaining femoral alignment. The goniometer placement followed standardized protocol: (1) stationary arm: parallel to the inter-anterior superior iliac spine line; (2) fulcrum: centered over the patella’s inferior pole; and (3) moving arm: aligned with the tibial crest. The terminal hip internal/external rotation angle was recorded at maximal displacement. The testing was performed bilaterally (three trials/limb; 30 s rest intervals) to assess hip external rotator function. Endpoints were: (1) passive resistance threshold: examiner-applied maximal torque without further displacement or (2) participant-reported endpoint: voluntary cessation due to pain/discomfort. Good inter-trial reliability was established (internal rotation test ICC = 0.76; external rotation test ICC = 0.89) [19].

6. Seated Trunk Rotation Test

**Figure 9:**
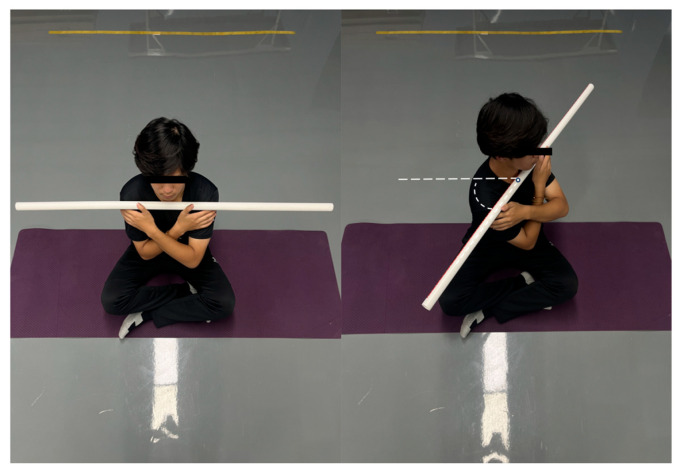
Seated trunk rotation test.

The participants assumed a cross-legged sitting position with hands grasping a stabilization bar at shoulder level. They performed maximal active trunk rotations to the left and right while maintaining pelvic stability. The goniometer was positioned at the vertex of the head to measure the horizontal rotation angle relative to the sagittal plane (Figure 9). Three trials per direction were conducted with 5 s rest intervals between repetitions. This protocol assessed active thoracolumbar rotation range of motion (ROM). Inter-trial reliability ranged from moderate to good (ICC = 0.59–0.82) [20].

7. Eyes-Closed Single-Leg Stance Test

**Figure 10:**
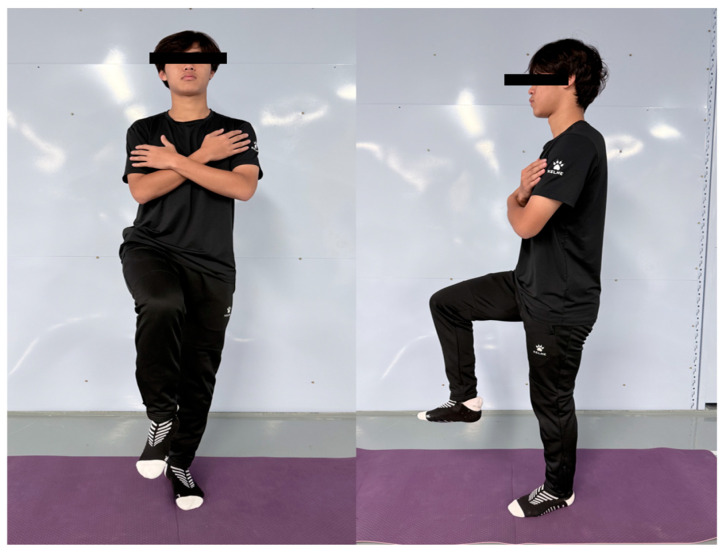
Eyes-closed single-leg stance test.

The participants assumed a single-leg stance on a firm surface (plinth) with hands crossed over the chest and the non-support leg flexed to 90° at the hip and knee (Figure 10). Upon achieving stable balance, the participants closed their eyes, at which point the examiner initiated timing. The test concluded upon either at (1) the loss of balance or (2) the completion of the 120 s—maximum duration. The procedure was performed bilaterally (one trial per limb) to assess static postural stability. The loss of balance was strictly defined by any of the following criteria: (1) displacement of the stance foot (lifting, sliding, or hopping); (2) contact of the non-support foot with the ground or stance limb; or (3) visual compensation (eye opening). Inter-trial reliability (intraclass correlation coefficient, ICC) was 0.72 [21].

8. Y-Balance Test (YBT)

**Figure 11:**
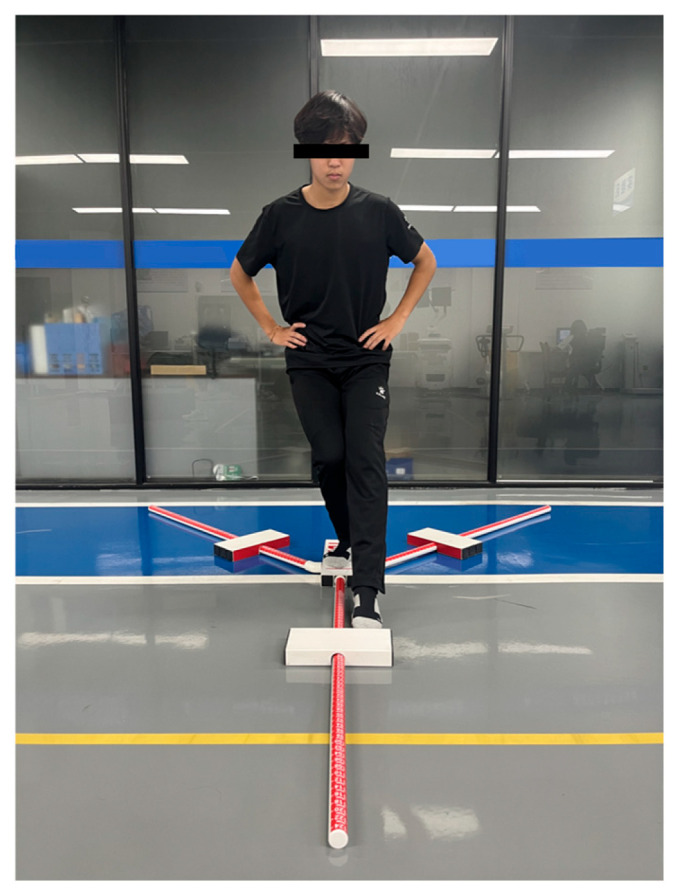
Y-balance test.

The participants assumed a unilateral stance on the central platform, with hands placed on the iliac crests and the stance foot positioned against the reference line. Using the free limb, the participants maximally displaced the reach indicator targets in three directions: anterior (ANT) (Figure 11), posteromedial (PM), and posterolateral (PL). Following each reach, the participants returned to the starting position under control. Three trials per direction were performed bilaterally, with 30 s rest intervals between sets. Reach distances were recorded to quantify dynamic postural stability. The trials were invalidated if any criterion was violated: (1) loss of heel contact with the central platform by the stance foot; (2) use of momentum/kicking motion to displace the target (non-controlled reach); or (3) ground contact by the reach foot during displacement. YBT demonstrated excellent inter-rater reliability (ICC = 0.85–0.93) across all three directions [22].

9. Back Extension Test

**Figure 12:**
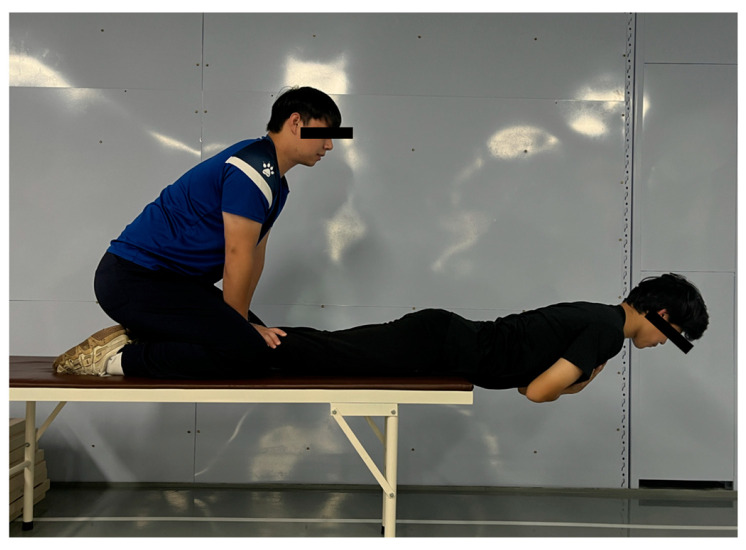
Back extension test.

The participants maintained a horizontal prone position with hands crossed over the chest, upper body suspended beyond the platform edge, and lower body fixed. Upon achieving proper positioning, the examiner initiated timing (Figure 12). The test terminated when either: (1) the participant failed to maintain horizontal alignment or (2) the 120 s maximum duration elapsed. Trunk endurance was quantified as time-in-position. The loss of horizontal alignment was objectively defined using a two-strike protocol: (1) first deviation: verbal warning issued when the participant’s trunk dropped below the horizontal plane; (2) second deviation: immediate test termination on subsequent failure. Inter-trial reliability (intraclass correlation coefficient, ICC) ≥ 0.77 [23].

### 2.4. Statistical Analysis

Prior to statistical analysis, the normality of all continuous variables was assessed using the Shapiro–Wilk test. The following variables were non-normally distributed: general joint laxity single-leg stance, back extension test, hip internal rotation, hip external rotation, and the posterolateral and posteromedial directions of the Y-balance test. For comparisons between collegiate and adolescent soccer athletes, the variables that did not meet the assumption of normality were analyzed using the Mann–Whitney U test. Independent-samples t-tests were applied to normally distributed variables to examine differences in physical functional characteristics. For within-group bilateral comparisons (left vs. right side), paired-samples t-tests were used for normally distributed data, while the Wilcoxon signed-rank test was performed for non-normally distributed variables. All statistical analyses were performed using SPSS, version 26 (SPSS Inc., Chicago, IL, USA). The level of statistical significance was set at *p* < 0.05.

## 3. Results

Table 2 shows basic parameters of college and adolescent athletes. There were significant differences in age (*p* < 0.001), training days per week (*p* < 0.001), training hours per day (*p* < 0.001), and years of soccer experience (*p* < 0.001) between college and adolescent athletes. No significant differences in other variables were found.

**Figure 13:**
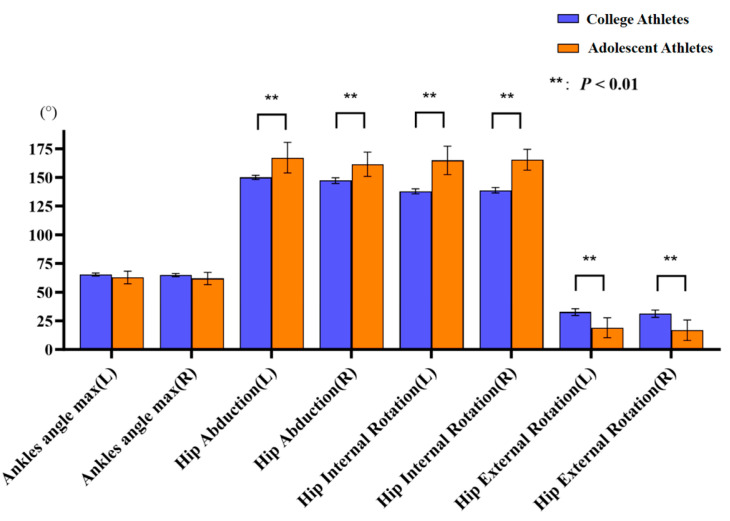
Comparisons of joint range of motion between college and adolescent athletes. Error bars indicate standard deviation.

College athletes showed significantly lower mean scores (1.25 ± 0.94 vs. 2.02 ± 1.07; *p* = 0.01) than adolescent athletes (Table 3). Regarding ROM, significant differences in bilateral hip abduction ROM (left: 150° ± 1.9° vs. 167.2° ± 13.3°; *p* < 0.01; right: 147.2° ± 2.5° vs. 161.5° ± 10.6°; *p* < 0.001), bilateral hip internal rotation ROM (left: 137.9° ± 2.1° vs. 164.8° ± 12.4°; *p* < 0.001; right: 138.8° ± 2.4° vs. 165.4° ± 9°; *p* < 0.001), and bilateral hip external rotation ROM (left: 32.6° ± 3° vs. 19° ± 8.7°; *p* < 0.01; right: 31.2° ± 3.2° vs. 16.7° ± 8.9°; *p* < 0.001) were identified between college and adolescent athletes (Figure 13). In addition, among adolescent athletes, significant side-to-side differences were observed in hip abduction ROM (left: 167.2° ± 13.3° vs. right: 161.5° ± 10.6°; *p* = 0.023).

**Figure 14:**
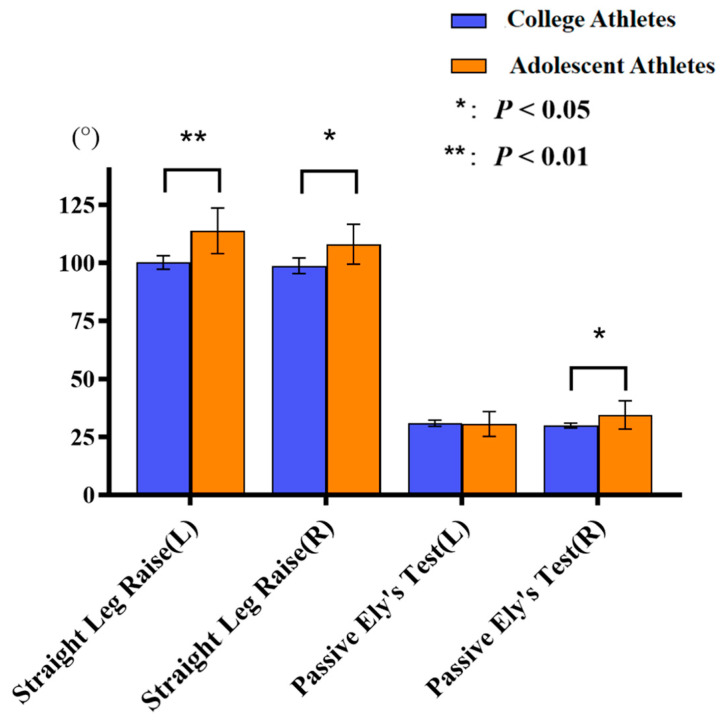
Comparison of SLR and passive Ely’s tests between college and adolescent athletes. Error bars indicate standard deviation.

College athletes exhibited significantly lower SLR degrees bilaterally (left: 100.1° ± 2.9° vs. 113.8° ± 9.8°; *p* < 0.01; right: 98.7° ± 3.3° vs. 108.0° ± 8.6°; *p* = 0.018) and significantly greater degrees on passive Ely’s test (29.9° ± 1.0° vs. 34.4° ± 6.1°; *p* < 0.01) compared to adolescent athletes (Figure 14). Among adolescent athletes, significant bilateral differences were observed in both SLR (113.8° ± 9.7° vs. 108.0° ± 8.5°; *p* < 0.01) and passive Ely’s tests (30.6° ± 5.3° vs. 34.5° ± 6.0°; *p* < 0.01).

Regarding balance ability, no significant differences were observed in static balance ability between college and adolescent athletes.

In YBT (Table 4), college athletes demonstrated significantly greater YBT reach distances in posterolateral direction of left leg (left: 127.06 ± 11.60 cm vs. 115.85 ± 9.50 cm; *p* < 0.01) and bilateral YBT posteromedial reach distances (left: 123.52 ± 10.90 cm vs. 116.68 ± 7.20 cm; *p* = 0.048; right: 121.11 ± 10.50 cm vs. 115.45 ± 7.00 cm; *p* = 0.026) compared to adolescents.

Among adolescent athletes, significant side-to-side differences were found in YBT posterolateral reach distances (115.84 ± 9.50 cm vs. 120.88 ± 6.20 cm; *p* < 0.01) with no other significant bilateral differences observed in this group.

College athletes exhibited significant bilateral differences in dynamic balance ability for the YBT anterior direction (68.33 ± 7.96 cm vs. 70.92 ± 8.20 cm; *p* = 0.018), while no significant side-to-side differences were detected in other directions.

**Table 4:** Comparisons of Y-balance tests between college and adolescent athletes.

		College Athletes	Adolescent Athletes
Anterior	Left leg (cm)	**68.33 ± 7.96 ^+^**	70.76 ± 6.72
	Right leg (cm)	70.92 ± 8.20	73.00 ± 7.32
Posterolateral	Left leg (cm)	**127.06 ± 11.60** *	115.85 ± 9.50
	Right leg (cm)	125.96 ± 8.87	**120.88 ± 6.18 ^+^**
Posteromedial	Left leg (cm)	**123.52 ± 10.90** *	116.68 ± 7.20
	Right leg (cm)	**121.11 ± 10.50** *	115.45 ± 7.00

Values are mean ± SD. * *p* values < 0.05 between collegiate and adolescent athletes. ^+^ *p* values < 0.05 between left and right legs. Bold indicates significant between-group differences.

**Figure 15:**
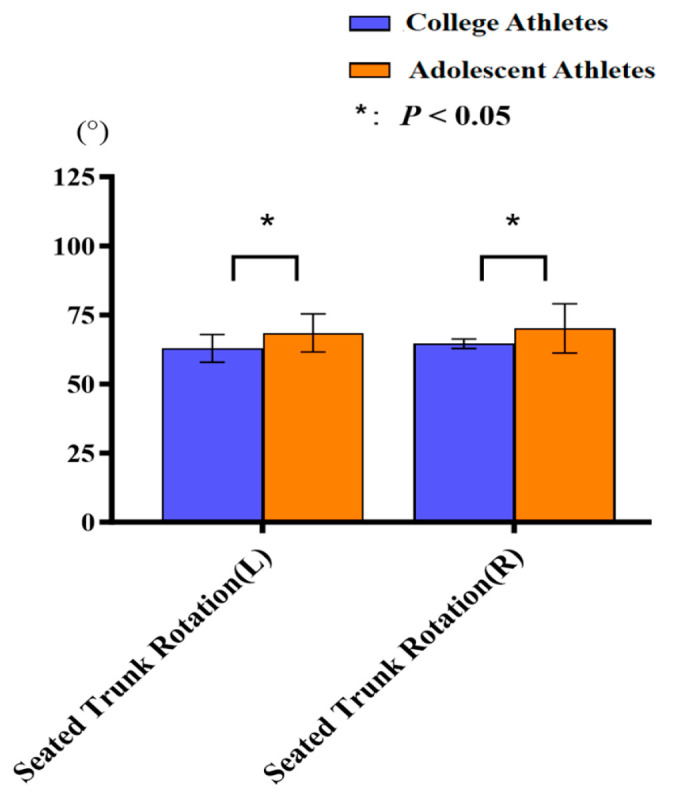
Comparison of seated trunk rotation between college and adolescent athletes. Error bars indicate standard deviation.

College athletes demonstrated significantly weaker trunk endurance times compared to adolescent athletes (96.60 s ± 5.00 s vs. 117.00 s ± 7.10 s; *p* < 0.01). Additionally, college athletes exhibited significantly weaker bilateral trunk rotation range of motion (left: 62.80° ± 1.70° vs. 68.50° ± 6.90°; *p* = 0.005; right: 64.60° ± 1.90° vs. 70.10° ± 8.90°; *p* = 0.034) (Figure 15).

## 4. Discussion

In this study, some vital findings were found: (1) adolescent athletes demonstrated significantly greater joint ROM and muscle flexibility compared to collegiate athletes; (2) significant differences in YBT existed between collegiate and adolescent athletes; and (3) contrary to expectations, adolescents displayed greater trunk endurance strength and larger trunk rotation ROM than collegiate athletes.

These physical functional divergences align with documented age-related patterns across sports disciplines: football/soccer players developed increased height, body mass, agility, and power output with maturation [24]; basketball players showed age-dependent gains in hamstring/quadriceps strength [25], vertical jump, and upper-body power [26]; rugby athletes under 16 exhibited comprehensive physical disparities across ages while under-20 cohorts showed primarily strength-dimension variations [27]; male tennis players demonstrated maturation-dependent progression in jump height and dynamic balance [28]; and baseball players developed accelerated sprint capacity and core power during growth [29]. This evidence substantiates the necessity for sport-specific functional assessment standards tailored to distinct age cohorts.

### 4.1. Joint Range of Motion

In this study, we confirmed that the adolescent athletes showed significantly greater joint ROM compared to the college athletes, aligning with past studies. A study on 72 youth soccer players across age groups demonstrated significantly greater joint laxity scores in younger cohorts [30]. These findings suggest progressive alterations in joint mobility during soccer players’ development, with younger athletes displaying particularly enhanced hip flexibility, which aligns with our findings. Further supporting this pattern, a study of 286 male youth soccer players (aged 10–19) documented an increasing prevalence of restricted knee flexion ROM with advancing age and maturation stage [19]. However, this study reported no significant bilateral asymmetry among athletes, contrary to our findings. Similar asymmetry patterns emerged in another study [11], which attributed observed hip asymmetries to repetitive predominant kicking/ball control actions in soccer.

### 4.2. Muscle Flexibility

In this study, compared to college students, adolescents exhibited significantly greater SLR and passive Ely’s test ROM, which means greater hamstring and quadriceps flexibility. Previous studies have consistently demonstrated greater flexibility in younger adolescent soccer players, aligning with our findings [30,31,32]. However, several studies have reported greater hamstring flexibility in older athletes [30,33], suggesting that the improvements in hamstring flexibility may represent an adaptive response to long-term participation in soccer. These findings differ from the findings of our study and may be attributable to differences in assessment methods, training phases, or athletic experience of the study populations. It is noteworthy that, although the adolescent athletes in this study had fewer total training years than the collegiate athletes, they reported significantly higher weekly training frequency and longer training duration per session. The observation that younger athletes with greater weekly training exposure demonstrated superior hamstring flexibility suggests that the observed flexibility profile may be a combined outcome of current training regimens and biological age rather than being determined by age alone. However, because other potential determinants of hamstring flexibility, such as injury history or habitual stretching routines, were not incorporated into the analytical framework, it cannot be concluded that a higher training volume directly affects hamstring flexibility. Future research incorporating a broader range of influencing variables is warranted to clarify the underlying relationships.

In addition, this study found significant bilateral asymmetries in both hamstring and quadriceps flexibility among adolescent athletes, whereas no such asymmetries were observed in the collegiate group. A previous study reported similar findings, indicating that younger athletes exhibited greater interlimb asymmetry compared with older athletes [34]. It should be noted that in our study, participants were strictly screened for limb dominance and history of injury to ensure that the measurements reflected functional status rather than acute trauma or pathological factors. The differences in muscular strength may significantly influence flexibility outcomes. The bilateral imbalances in hamstring and quadriceps flexibility among adolescent athletes could potentially originate from strength differentials between dominant and non-dominant limbs. Soccer, a sport requiring frequent unilateral execution of technical actions with the preferred limb, tends to exacerbate bilateral muscular asymmetry. During adolescents’ rapid skeletal maturation phases, high-intensity training regimens may further amplify these strength asymmetries. Post-adolescence, diminished growth velocity coupled with the deliberate implementation of bilateral balance training in adulthood likely promotes greater interlimb symmetry. This developmental trajectory potentially explains the pronounced bilateral asymmetries observed in adolescent athletes versus the enhanced bilateral balance demonstrated by collegiate athletes. However, since individual playing positions (e.g., midfielders, defenders, or strikers) were not factored into the subgroup analysis, these findings should be regarded as descriptive functional observations rather than definitive evidence of training-induced adaptations.

### 4.3. Balance Capacity

Balance capacity is primarily categorized into static and dynamic components. Regarding static balance, this study detected no significant differences between adolescent and collegiate athletes, contrasting with multiple prior studies reporting superior static balance in younger athletes [35,36]. Previous research has demonstrated that postural sway during balance testing varies across age groups, and that older athletes tend to exhibit reduced single-leg stance time compared with younger athletes [37]. Maintaining static equilibrium demands fewer functional resources, and younger athletes, typically characterized by lower body mass and stature, possess inherent advantages in sustaining stable postures [38]. In this study, no significant differences were observed between the two groups in anthropometric characteristics (height, body mass, and BMI), suggesting comparable physiological conditions for maintaining static balance, which may explain the absence of between-group differences in static balance performance.

In contrast, dynamic balance engages substantially more complex motor systems [39]; this requires athletes to possess more refined and stable neuromuscular control, which suggests that accumulated training years and the resulting neuromuscular refinement in the collegiate group may be more significant contributors to dynamic stability than chronological maturation alone. This finding was well-substantiated, with older football/soccer athletes exhibiting enhanced unilateral stability (evidenced by greater maximum reach distances in YBT) [36,40,41]. Our study also found that collegiate athletes achieved significantly greater reach distances in the posterior directions of the YBT. Dynamic balance is modulated by multifactorial influences, including age, height, body mass, athletic proficiency, and training experience [39]. Athletes in the older group typically undergo longer periods of systematic training, which may result in more extensive neuromuscular adaptations and, consequently, enhance their ability to maintain balance during sport-specific tasks.

Furthermore, this study observed interlimb asymmetry in the Y-balance test (YBT) across both the adolescent and collegiate athletes. This suggests that bilateral imbalances in dynamic stability may manifest early in the elite soccer pathway and persist into collegiate levels. Previous research has suggested that athletes who consistently rely on a dominant lower limb to perform technical movements, such as kicking or pivoting, may develop unilateral neuromuscular adaptations, thereby increasing the likelihood of interlimb asymmetry [42]. This may partially explain the findings of the present study. While some studies suggested that this relationship may be influenced by cumulative training years [34], our findings indicate that such functional asymmetry in dynamic balance is a common characteristic among elite soccer players regardless of the age group. Consistent with our overall approach, as participants were screened for injury and limb dominance, these asymmetries reflect functional status; however, without stratification by playing position, they remain descriptive observations of the elite athlete profile.

### 4.4. Trunk Functional Capacity

Contrary to the initial hypotheses, adolescent athletes demonstrated better trunk endurance compared to collegiate athletes, a finding inconsistent with previous studies. Generally, enhanced trunk endurance in older athletes is attributed to advanced physical maturation, refined control mechanisms, and superior stability maintenance capabilities [43,44,45,46]. It is noteworthy that in this study, no significant differences were observed between the two cohorts in height, body mass, or BMI, suggesting that these performance discrepancies are not attributable to the overall body size. We speculate that the observed differences may instead stem from variations in task familiarity, participant motivation, or sport-specific training emphases at different competitive stages. However, as this study did not account for these specific confounding variables within its cross-sectional design, these results are presented for descriptive purposes only. They should be interpreted cautiously and are intended to provide a basis for more controlled, longitudinal investigations in the future. Additionally, aligning with our expectations, adolescents exhibited greater trunk flexibility. In line with the existing literature, larger trunk rotation ranges were found among younger athletes relative to older athletes [47].

### 4.5. Limitations

Although this study provided valuable insights into age-related differences in physical functional characteristics among soccer players, several limitations should be acknowledged.

First, this study is characterized by the absence of biological maturation indicators (e.g., skeletal age or peak height velocity). In comparing mid-adolescent and young adult athletes, chronological age alone is an insufficient proxy for developmental status, as biological maturation significantly influences physical function. Consequently, the observed functional differences cannot be definitively attributed to age-related change but rather reflect combined developmental and stage-specific characteristics.

Second, although testing procedures were strictly standardized, the temporal and environmental heterogeneity between the two cohorts must be acknowledged. The assessments were conducted at different institutions and during different phases of the training year. Consequently, seasonal factors, including training periodization (e.g., pre-season vs. in-season), accumulated physical fatigue, and varying competitive demands, were not synchronized between the groups. These temporal variables may have introduced variance in functional outcomes such as flexibility and dynamic balance, potentially affecting between-group comparability.

Third, this study primarily employed functional tests to conduct a cross-sectional comparison and description of physical functional characteristics. It did not incorporate complementary approaches from sports medicine or biomechanics to further examine the underlying mechanisms contributing to these functional differences. Consequently, the findings remain largely at a descriptive level and are insufficient to explain the neuromuscular control, structural, or mechanical adaptations underlying age-related differences.

Fourth, the sample size of this study was relatively limited, and only two age groups, adolescents and collegiate athletes, were included. The relatively narrow age range may restrict the representativeness and generalizability of the findings and may not fully capture the overall trajectory of functional changes across age and training experience. In addition, the study included only male participants; therefore, the findings cannot be generalized to female soccer players, who may exhibit distinct functional characteristics due to sex-specific physiological and biomechanical differences.

Fifth, although all participants were strictly screened for limb dominance and prior injury history to ensure functional integrity, this study did not further stratify participants based on playing position. Given that positional demands in soccer significantly influence joint range of motion, muscle flexibility, and dynamic balance, the absence of position-specific stratification may have partially obscured subtle functional nuances within the cohorts.

Future research should consider expanding the sample size and age range, integrating sports medicine and biomechanical assessment techniques and implementing more refined participant stratification. Such approaches would allow for a more comprehensive and mechanistic understanding of age-related differences in physical functional characteristics among soccer players.

## 5. Conclusions

This study indicates that significant differences exist between Chinese elite college and adolescent male soccer athletes in physical function indicators. Overall, adolescent athletes are characterized by superior overall mobility and flexibility, encompassing greater joint range of motion and muscle extensibility, alongside higher trunk functional capacity. Conversely, collegiate athletes exhibit significantly greater dynamic balance stability. These findings demonstrate that elite collegiate athletes possess different functional characteristics compared to adolescent athletes, suggesting that training focuses should be tailored to the specific functional characteristics of each cohort. However, it should be noted that this study included only male athletes; therefore, the applicability of these findings to female soccer athletes remains uncertain and warrants further investigation in more diverse populations.

## Figures and Tables

**Table 1:** Screening criteria.

Inclusion Criteria	Exclusion Criteria
1. No history of musculoskeletal injury within the 3 months preceding the study;	1. Failure to provide legible/complete questionnaire responses;
2. No participation in strenuous exercise within the 24 h prior to testing;	2. Failure to complete the functional movement screening test.
3. A minimum of 5 years of systematic football training experience;	
4. Engagement in football training sessions ≥5 times per week.	

**Table 2:** Basic parameters of collegiate and adolescent athletes.

Variable	College Athletes (*N* = 20)		Adolescent Athletes (*N* = 30)		*p* Value
	Ave	SD	Ave	SD	
Age (yrs)	20.40	0.42	15.77	1.10	**<** **0.001**
Height (cm)	180.35	1.16	179.10	5.99	0.45
Weight (kg)	70.85	1.55	67.70	8.81	0.19
BMI (kg/m^2^)	21.77	1.80	21.03	1.85	0.17
Training days per week	5.75	0.79	6.5	0.57	**<0.001**
Training hours per day	1.98	0.41	2.6	0.45	**<0.001**
Years of soccer experience	11.25	2.88	7.77	1.85	**<0.001**

Bold *p*-values indicate significant between-group differences.

**Table 3:** General joint laxity score.

	College Athletes (*N* = 20)		Adolescent Athletes (*N* = 30)		
**General Joint Laxity Score**	Ave	SD	Ave	SD	*p* value
	1.25	0.94	2.02	1.07	**0.01**

Bold *p*-values indicate significant between-group differences.

## Data Availability

The datasets used and/or analyzed during the current study are available from the corresponding author on reasonable request.
